# Prospects of Food Taxes for Planetary Health: A Systematic Review of Modeling Studies

**DOI:** 10.1093/nutrit/nuae111

**Published:** 2024-09-16

**Authors:** Ágota Mészáros, Norbert Dósa, Anna Péterfi, Krisztián Horváth, Zsófia Szarvas, Jeremiás M Balogh, Balázs Munkácsy, Zoltán Vokó

**Affiliations:** Department of Public Health, Semmelweis University, Budapest, Hungary; Center for Health Technology Assessment, Semmelweis University, Budapest, Hungary; Department of Public Health, Semmelweis University, Budapest, Hungary; Department of Public Health, Semmelweis University, Budapest, Hungary; Department of Public Health, Semmelweis University, Budapest, Hungary; Department of Public Health, Semmelweis University, Budapest, Hungary; Department of Agricultural Economics, Corvinus University of Budapest, Budapest, Hungary; Budapest Institute for Policy Analysis, Budapest, Hungary; Doctoral School of Education, University of Szeged, Szeged, Hungary; Center for Health Technology Assessment, Semmelweis University, Budapest, Hungary; Syreon Research Institute, Budapest, Hungary

**Keywords:** planetary health, public health nutrition, modeling study, food tax, diet change

## Abstract

**Objectives:**

The aim of this study was to analyze the modeling methodologies of fiscal policies on food with health or environmental outcomes.

**Background:**

Evidence suggests that fiscal policies on food can contribute to addressing the growing burden of noncommunicable diseases and climate change. These policies should be modeled in advance to see the implications for the environment and health.

**Methods:**

A systematic review was conducted of studies that modeled fiscal policies on the food groups targeted by the EAT-Lancet Commission and examined their health or environmental outcomes. The Scopus and PubMed databases were searched on November 30, 2021. The records were double-screened and data on modeling methods were extracted from the included studies.

**Results:**

A total of 55 studies were included in the review. The most frequently modeled interventions were fruit and vegetable subsidies (n = 19) and carbon taxes on food (n = 17). One study also included a consumer education campaign to enhance the effect of fiscal policy. The outcomes are highly sensitive to consumption change and price elasticities. None of the studies modeled the health effects of environmental outcomes.

**Conclusions:**

A model that covered all the relevant aspects of the issue was not found. Some parts were missing from all the included models. It is advisable to model the stability of the amount of diet consumed, either by keeping the amount of food in the diet stable or by taking a more conservative approach and keeping the consumed calories stable. It is preferable to keep the included diseases and environmental boundaries broad to have more valid outcome estimates on this complex issue. A more comprehensive understanding of fiscal policies would allow us to better anticipate the impact of our actions and inactions and thus could lead to more sophisticated measures taken by policymakers.

**Systematic Review Registration:**

PROSPERO registration no. 2022 CRD42022291945/

## BACKGROUND

The rapid increases in the prevalence of noncommunicable diseases and environmental change are among the leading global challenges of the 21st century, both from health and economic perspectives.^1^ An unhealthy diet is a major risk factor for noncommunicable diseases worldwide: diet-related disease burden accounted for 14% of all deaths^2^ and 7.4% of all disability-adjusted life years^3^ in 2019. Considering environmental change, agriculture is responsible for approximately 34% of all greenhouse gas (GHG) emissions^4^ and food production itself is the leading cause of biodiversity loss,^5^ which, like climate change, has dire implications.^6^

Climate change and biodiversity loss are damaging not only to the environment but also to human health.^6^^,^^7^ Many natural disasters can be linked to environmental change. Emerging infectious diseases and heat waves, for example, are causing increasing disease burdens and numbers of deaths.^6^^,^^8^ There is growing evidence that a shift from an animal-based diet toward a plant-based diet can contribute to addressing both of these problems.^9^

According to the EAT-Lancet Commission, we can “fill two needs with one deed”^10^: by altering our diets in a culturally accepted way, we can help ourselves and the planet. Specifically, a shift, at a societal level, in our eating habits to align them more closely to the EAT-Lancet Commission’s Planetary Health Diet, which is a plant-based diet, would be beneficial for people and the planet. These dietary changes are all in line with the goals 3 and 13 of the United Nations’ sustainable development goals, namely, good health and well-being and climate action, respectively.^11^

Altering prevailing dietary habits, however, is far from easy. The World Health Organization described the primary barriers that need to be mentioned: the globally higher price of nutritious food, culturally-engrained dietary habits, and the current food environments that are pushing societies toward consumption of low-cost, energy-dense foods.^9^

When thinking about changing consumption, policymakers have 3 main ways to choose from: regulatory measures, such as bans or modification of product standards; fiscal measures, like food taxes or subsidies; and informational measures, such as mandatory labeling or consumer campaigns.^12^ Informational measures are encouraged by the industry because they place responsibility on the consumer (however, they usually do not affect the lower socioeconomic groups much),^13^^,^^14^ and regulatory measures are mostly used when there is an acute threat to health.^15^ Fiscal measures directly address the externalities connected to specific food groups; they reach the majority of the population and could even lessen the health disparities between socioeconomic groups, as opposed to the information measures, which might make health inequalities larger.^14^ With properly targeted fiscal interventions on food, it is easier for consumers to choose a healthier and more environmentally friendly diet.

Before the implementation of public health interventions, it is crucial to simulate them in a model to help decision-making by estimating all relevant health and environmental consequences and costs. In the case of complex interventions, especially in environmental or public health, these long-term consequences can only be explored via modeling.^16^ Because this topic is relatively new, there are no clear guidelines or protocols developed yet on how to model the effects of these fiscal interventions on multiple outcomes, such as human health and the environment.

Reviews have been published on similar topics, but their aim was to compare the results of these models^17^ and not to analyze the methods, or they only analyzed the models with health outcomes^18^ but did not consider the environmental outcomes. The recent World Health Organization Policy Brief about fiscal measures for healthy diets was designed to support policymakers in implementing food taxes,^19^ but it did not give guidance on modeling the effects of these taxes. The aim of this systematic literature review was to scrutinize models that estimated the environmental and health effects of food taxes and subsidies. We defined modeling as a simplified representation and estimation of the effect of fiscal policies on food on health, environment, and costs.

## METHODS

Two databases (Scopus and PubMed) were searched on November 30, 2021, and data about the models were extracted from the selected publications. The search string was made in line with the Population, Intervention, Comparison, Outcomes, and Study design (PICOS) framework (Table 1), with keywords and Medical Subject Heading terms connected to different food groups, fiscal interventions, the model, and outcomes, including environmental and health outcomes. The keywords within groups were connected with OR Boolean operators, and all 4 keyword groups were connected with AND Boolean operators. The search string can be found in Supporting Information S1.

**Table 1.** PICOS Criteria for Inclusion of Studies

Criteria	Determinants
Participants	Whole population or selected population groups in countries or regions
Interventions	Fiscal policy on a specific food group from the EAT-Lancet Commission Report: Meat, dairy, eggs, fish, vegetables, fruit, whole grains, legumes, nuts
Comparisons	No new intervention
Outcomes	Health or environmental outcome
Study design	Models that estimate the effects of the fiscal policy on food

Before title and abstract screening, we conducted a pilot on 70 records and refined our inclusion and exclusion criteria (Table 2), which were thoroughly explained in a training for the reviewers. Title and abstract screening of records was supported by the ASReview tool,^20^ a software program that uses machine learning to order the records, putting the most relevant first, based on the previous inclusion/exclusion decisions made by the researcher. The primary screener screened all titles and abstracts, and 3 secondary screeners independently screened one-third of the records each. ASReview recommends screening 40% of records to be sure one does not miss any important records. For accuracy, we screened 50% of the records. After that, conflict resolution was reached with the help of a third researcher.

**Table 2.** Hierarchical Exclusion Criteria for the Title and Abstract Screening and Full-Text Screening of the Records

Title and abstract screening exclusion criteria	Full-text screening exclusion criteria
––	Full text is not available
No human study subjects (eg, only animals or bacteria)	No human study subjects (eg, only animals or bacteria)
No food product studied (eg, only alcohol, tobacco)	No food product studied (eg, only alcohol, tobacco)
No food product of interest studied (including animal products [meat, dairy, eggs, fish], and healthy plant products [eg, vegetables, fruit, whole grains, legumes, nuts]; excluding only sugar-sweetened beverages, salt, junk food)	No food product of interest studied (including animal products [meat, dairy, eggs, fish], and healthy plant products [vegetables, fruit, whole grains, legumes, nuts]; excluding only sugar-sweetened beverages, salt, junk food)
No fiscal policy studied targeting food product groups (eg, only health promotion campaign)	No fiscal policy studied targeting food groups (eg, only health promotion campaign)
No modeling performed on the effects of the fiscal policy of food	No modeling performed on the effects of the fiscal policy of food
No health or environmental outcome studied –– ––	No health or environmental outcome studied Studies without assessment of the environmental impact of the fiscal policy or health outcome of the fiscal policy If review: exclude and check if in the reviewed studies are new relevant studies (via title and abstract screening)
Included	Included

Additional records were found through a gray literature search and citation search of reviews identified in the list of records. A gray literature search was conducted on the websites Dart-Europe^21^ and OpenDissertations^22^ on February 22, 2022; the search details can be found in Supporting Information S1. We also included the relevant reviews in title and abstract screening and checked the references in the included reviews, after which the titles and abstracts of identified records were screened.

**Figure 1.**
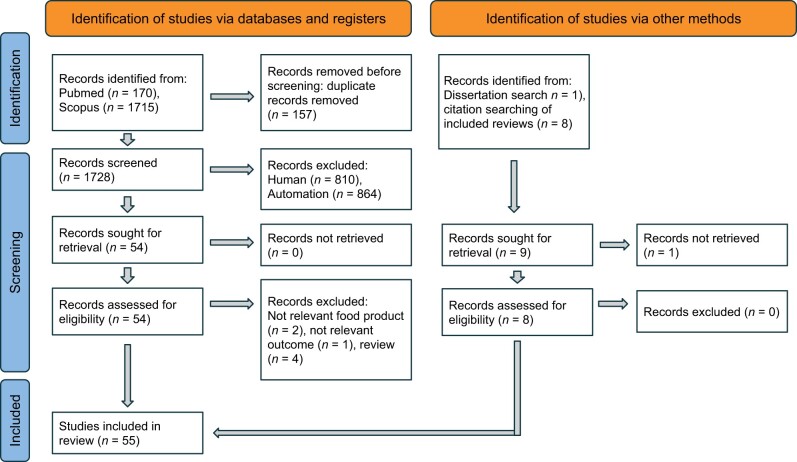
PRISMA Flowchart

The full texts of the collected articles were read by 2 researchers independently, followed by conflict resolution. The whole screening process is shown in a PRISMA^23^ flowchart in Figure 1, and the PRISMA 2020 checklists can be found in Supporting Information S2 and S3.

Data extraction was done in Google Sheets by 1 reviewer and verified independently by a second. The data extraction table was made iteratively, adding more data item columns as new and relevant data came up in the records, as well as by consulting with coauthors with different areas of expertise. In most cases, the data on some items (eg, price elasticities) were not available from the original article, so we looked for them in previous literature cited in the original article.

The study variables were sorted into the following groups: general data about the analysis and the model, considered cost items, consumption change, health model details, and environmental model details. Because of the large variety of interventions and differences in the characteristics of the studies, we did not compare the results of the analyses of the included studies. The review is registered in the PROSPERO database (registration no. 2022 CRD42022291945).

## RESULTS

A total of 1885 records were retrieved: 1715 from Scopus, 170 from PubMed, and 9 from grey literature. After title and abstract screening and full-text screening, 55 records were included in the review; the exact numbers in different screening stages are indicated in Figure 1.

Selected data items from the 55 articles^24–78^ are listed in Table 3, and a table in the form of a full data extraction table with all details is provided in spreadsheet in Supporting Information S4.

**Table 3.** Characteristics of the Included Studies

First author, year	Intervention arms	Country or region	Perspectives of the model	Cross-price elasticities	Stability of consumed food	Diseases included	Environmental outcomes
Dogbe, 2021^24^	Subsidy on F&V needed for 10% increase in F&V consumption (F 21%, V 13%)	UK	Environment	Yes	No	NA	CO_2_-eq emissions
Ritchie, 2018^25^	Subsidy: 5% to 75% reduction in the average 2015 market price of standard Quorn products	40 high-income countries in Europe, North America, Australia, New Zealand, South Africa, Asia	Environment, healthcare	Yes: for meat products	No	Coronary heart disease, stroke, cancer, obesity	CO_2_-eq emissions
Markandya, 2016^26^	Combined taxes and subsidies to reach optimal diet	Spain	Environment, consumers, government	Yes	No	NA	CO_2_-eq emissions
Springmann, 2018^27^	Tax on red and processed meat, different tax rates depending on the country income (low, middle, high)	149 world regions	Healthcare, society, government, environment	Yes	No	Coronary heart disease, stroke, colorectal cancer, type 2 diabetes, weight (underweight, obesity)	CO_2_-eq emissions
Ni Mhurchu, 2015^28^	20% subsidy on F&V 20% tax on main sources of dietary sodium 20% tax on main sources of saturated fat A combination of these 3 20% tax on major food group contributors to carbon emissions	New Zealand	Healthcare	Yes	Yes: calorie stability in F&V subsidy scenario, no in other scenarios	Cardiovascular disease, diabetes, diet-related cancers, obesity, kidney disease	NA
Lee, 2019^29^	30% subsidy on F&V, by EBT card 30% subsidy on F&V, whole grains, nuts/seeds, seafood, and plant oils, by EBT card	USA	Healthcare, society, government	No	No	Coronary heart disease, stroke (ischemic, hemorrhagic), type 2 diabetes	NA
Mozaffarian, 2018^30^	30% subsidy on F&V, by EBT card 30% subsidy on F&V with restriction of SSBs, by EBT card 30% subsidy on F&V, nuts, whole grains, fish, and plant-based oils; 30% tax on SSBs, junk food, and processed meats, by EBT card	USA	Healthcare, government	No	No	Coronary heart disease, stroke, type 2 diabetes, obesity	NA
Cobiac, 2017^31^	1.37 AUD tax on 100 g of saturated fat 0.30 AUD tax on 1 g of sodium 0.47 AUD tax on 1 L SSB 0.14 AUD subsidy on 100 g of F&V Tax on sugar: 0.94 AUD tax on 100 mL of ice cream; 0.85 AUD tax on 100 g of sugar	Australia	Healthcare, government, consumers	Yes	Yes: calorie and weight of food stability in sensitivity analysis	Ischemic heart disease, ischemic stroke, cancer (colon, lung, stomach, esophagus, breast, endometrium, kidney, thyroid), systolic blood pressure, hypertensive heart disease, type 2 diabetes, osteoarthritis, obesity	NA
Peñalvo, 2017^32^	10% tax on SSB, red and processed meats, 10% subsidy on F&V, nut/seeds, whole grains 30% tax on SSB, red and processed meats, 30% subsidy on F&V, nut/seeds, whole grains	USA	Healthcare	No	No	Coronary heart disease, stroke, type 2 diabetes, obesity	NA
Broeks, 2020^33^	15% tax on meat 30% tax on meat 10% subsidy on F&V	The Netherlands	Healthcare, society, consumers, government, environment	No	No	Type 2 diabetes, stroke, coronary heart disease, colorectal cancer, lung cancer	CO_2_-eq emissions, acidification (SO_2_-eq), saltwater eutrophication (N-eq), freshwater eutrophication (P-eq), land use
Basu, 2013^34^	Ban on SSB, using SNAP dollars 22% tax on SSB, using SNAP dollars 30% subsidy on F&V, using SNAP dollars Overall increase of SNAP budget Overall increase of SNAP budget, and food intake pattern changes	USA	Healthcare, government	Yes	No	Cardiovascular disease, type 2 diabetes, obesity	NA
Jansson, 2018^35^	Tax on meat and dairy products: 16 EUR, 60 EUR, and 290 EUR/ton of CO2-eq	EU	Environment, government, consumers	Yes	No	NA	CO_2_-eq emissions
Mytton, 2007^36^	17.5% tax on main sources of dietary saturated fats 17.5% tax on less healthy foods by SSCg3d scores^a^ 17.5% tax on food groups for optimal outcome	UK	Healthcare, consumers	Yes	No	Cardiovascular disease (stroke, ischemic heart disease)	NA
Wilde, 2018^37^	Subsidy on F&V, nuts, whole grains: 10% and 30% Subsidy on F&V, nuts, whole grains; tax on SSB, processed meats: 10% and 30% 10% subsidy + 30% additional SNAP subsidy on F&V, nuts, whole grains 10% subsidy + 30% additional SNAP subsidy on F&V, nuts, whole grains; 10% tax on SSB, processed meats	USA	Healthcare	No	No	Coronary heart disease, stroke, diabetes, obesity	NA
Briggs, 2013^38^	2.72 GBP tax/ton of CO2-eq on food and drink with above average emissions 2.72 GBP tax/ton of CO2-eq on food and drink with above average emissions + subsidy for below average emissions until revenue neutral	UK	Healthcare, environment, government	Yes	Yes: calorie stability in sensitivity analysis	Coronary heart disease, stroke, cancer, obesity	CO_2_-eq emissions
Tönnies, 2021^39^	50% tax on red meat 50% tax on SSB 50% tax on tobacco A combination of these	Germany	Healthcare	No	No	Type 2 diabetes, obesity	NA
Magnus, 2016^40^	20% subsidy on all fruit 20% subsidy on fresh vegetables 20% subsidy on all vegetables 20% subsidy on F&V 20% subsidy on diet drinks and water 20% subsidy on all F&V, diet drinks and water	Australia	Healthcare, government	Yes	Yes: weight of food stability	Ischemic heart disease, stroke, hypertensive heart disease, type 2 diabetes, breast and colon cancer, obesity	NA
Nnoaham, 2009^41^	17.5% tax on main sources of dietary saturated fats 17.5% tax on less healthy foods by WXYfm scores 17.5% tax on less healthy foods by WXYfm scores + subsidy on F&V: 17.5% and 32.5%^c^	UK	Healthcare, consumers, government	Yes	No	Cardiovascular disease (stroke, coronary heart disease), cancer, obesity	NA
Caillavet, 2016^42^	20% tax on all food groups with animal content 20% tax on some food groups with animal content: beef, cooked meats, animal fats, cheese, prepared mixed meals	France	Consumers, environment	Yes	No	NA	CO_2_-eq emissions, air acidification (SO_2_-eq), water eutrophication (N-eq)
Holm, 2013^43^	Halved VAT on F&V; 8 DKK tax on 1 kg of fat; 5.6 DKK tax on 1 kg of sugar	Denmark	Healthcare, consumers, government	Yes	No	Ischemic heart disease, ischemic stroke, colorectal cancer, lung cancer, breast cancer	NA
Blakely, 2020^44^	20% subsidy on F&V + tax on saturated fat, sugar, and salt until revenue neutral (these 3 together and separately as well) Tax on saturated fat, sugar and salt (separately, the same amount of tax as above) 8% tax on nonessential energy-dense foods	New Zealand	Healthcare, government, consumers	Yes	No	Coronary heart disease, stroke, osteoarthritis, diabetes, obesity, multiple cancers: endometrial, head and neck, kidney, liver, lung, esophageal, pancreatic, stomach, thyroid, colorectal, breast, ovarian, and gallbladder	NA
Schönbach, 2019^45^	Tax on processed meat: 4%, 11.2%, 18.5%, 33.3%	Germany	Healthcare	Yes	No	Ischemic heart disease, diabetes, colorectal cancer	NA
Saha, 2021^46^	Tax on products rich in: Salt: 11.61% and 20% Saturated fat: 11.61% and 20% Saturated fat 11.61% and 20%; subsidy on F&V 10.71% and 20% Saturated fat and salt 11.61% and 20%; subsidy on F&V 10.71% and 20% Subsidy on F&V: 10.71% and 20%	Sweden	Healthcare, consumers	Yes	No	Coronary heart disease, stroke, obesity, diet-related cancers	NA
Gren, 2019^47^	Tax on beef: based on GHG emission, using different weights; with and without correction for double tax Tax on beef: Based on GHG emission, using GWP100; with and without correction for double tax	Sweden	Environment	Yes: for pork and chicken	No	NA	CO_2_-eq emissions
Moberg, 2021^48^	GHG tax, using GWP100, on: All food Animal products Beef Monogastric meat and eggs GHG tax on all food, based on weighting of several environmental impacts 11.61% tax on animal products 5.36% subsidy on F&V and cereals A combination of the last 2	Sweden	Environment	Yes	No	NA	CO_2_-eq emissions, cropland use, pasture use, N application, P application, consumptive freshwater use, terrestrial extinction rate, NO_x_ emissions, NH_3_ emissions, pesticide use, N_2_O emissions
Edjabou, 2013^49^	Tax on all foods: 0.26 DKK and 0.76 DKK/kg of CO_2_-eq Tax on all foods: 0.26 DKK and 0.76 DKK/kg of CO_2_-eq + lowering the VAT for all foods until revenue neutral	Denmark	Environment, consumers	Yes	No	NA	CO_2_-eq emissions
Rahkovsky, 2013^50^	10% tax on processed foods 10% tax on regular-fat milk products 10% subsidy on vegetables 10% subsidy on whole-grain products	USA	Healthcare, government, society	No	No	Cardiovascular disease, stroke	NA
Bonnet, 2018^51^	Tax on all animal products: 56 EUR and 200 EUR/ton of CO2-eq Tax on beef, veal, lamb, sheep: 56 EUR and 200 EUR/ton of CO2-eq Tax on all beef: 56 EUR and 200 EUR/ton of CO2-eq	France	Environment, consumers, government	Yes	No	NA	CO_2_-eq emissions, air acidification (SO_2_-eq), water eutrophication (N-eq)
Kim, 2019^52^	10% tax on processed meat Warning label on processed meat	USA	Healthcare, government, society	No	No	Colorectal and stomach cancer	NA
Choi, 2017^53^	30% subsidy on F&V for SNAP participants, limited to 60 USD/person/month	USA	Healthcare, government	Yes: for refined grains and 100% fruit juice	No	Obesity, type 2 diabetes, myocardial infarction, stroke	NA
Wirsenius, 2011^54^	60 EUR tax/ton of CO_2_-eq on animal food products, except fish and seafood	EU	Environment	Yes	No	NA	CO_2_-eq emissions, land use change
Vandenberghe, 2018^55^	Tax on all food products and fuels: 30 EUR, 45 EUR, and 60 EUR/ton of CO_2_-eq	Belgium	Environment, healthcare, society	Yes	No	Cardiovascular disease, cancer, type 2 diabetes	CO_2_-eq emissions
Kehlbacher, 2016^56^	2.841 GBP tax/ton of CO_2_-eq on all foods 2.841 GBP tax/ton of CO_2_-eq on foods with above average emissions	UK	Environment, government	Yes	No	NA	CO_2_-eq emissions
Caillavet, 2019^57^	Tax on all foods: 56 EUR and 140 EUR/ton of CO_2_-eq Tax on highest emitting, animal protein–rich foods: 56 EUR and 140 EUR/ton of CO_2_-eq Tax on highest emitting, animal protein–rich foods: 56 EUR and 140 EUR/ton of CO_2_-eq + subsidy on fresh F&V and starchy vegetables (including legumes) until revenue neutral	France	Environment, consumers, government	Yes	No	NA	CO_2_-eq emissions, air acidification (SO_2_-eq), water eutrophication (N-eq)
García-Muros, 2017^58^	Tax on all foods: 25 EUR and 50 EUR/ton of CO_2_-eq 50 EUR tax/ton of CO_2_-eq on all foods, except F&V, cereals, milk	Spain	Environment, consumers	Yes	No	NA	CO_2_-eq emissions
Zech, 2019^59^	50 USD tax/ton of CO_2_-eq on all food	EU	Environment	No	Yes: calorie stability	NA	CO_2_-eq emissions
Springmann, 2018^60^	23 AUD tax/ton of CO_2_-eq on all food	Australia	Environment, healthcare, government	Yes	No	Weight-related risk (underweight, overweight, obese), coronary heart disease, stroke, type 2 diabetes, diet and weight-related cancers (colon, rectum, mouth, oropharynx, esophagus, stomach, trachea, bronchus, lung, aggregate cancer), all other causes	CO_2_-eq emissions
Pinho-Gomes, 2021^61^	10% subsidy on F&V 30% subsidy on F&V for low-income households Social marketing campaign to increase F&V consumption	England	Healthcare, government	No	No	Type 2 diabetes, ischemic heart disease, cerebrovascular disease, cancer (bladder, stomach, breast, colorectum, esophagus, trachea and lung, pharynx, larynx, oral cavity)	NA
Briggs, 2016^62^	2.86 GBP tax/ton of CO_2_-eq on food above average emissions 2.86 GBP tax/ton of CO_2_-eq on food above average emissions + subsidies on food below average emissions until revenue neutral Both scenarios also combined with a 20% tax on SSB	UK	Environment, healthcare, government	Yes	Yes: calorie stability for food (not for drinks)	Cardiovascular disease, diabetes, diet-related cancers, COPD, kidney disease, liver disease	CO_2_-eq emissions
Magnus, 2018^63^	20% discount on F&V, artificially sweetened soft drinks, and bottled water 20% discount on F&V, artificially sweetened soft drinks and bottled water + consumer education	Australia	Healthcare, government	Yes	No	Ischemic heart disease, stroke, hypertensive heart disease, type 2 diabetes and its complications, colon cancer, obesity	NA
Revoredo-Giha, 2018^64^	Tax on beef and veal: 20% or different carbon prices^b^ Tax on all meat and eggs: 20% or different carbon prices^b^ Tax on all animal products: 20% or different carbon prices^b^ Tax on all food: 5% to 30% on different food groups or different carbon prices^b^ All scenarios above also combined with subsidy on all food until revenue neutral	UK	Environment	Yes	No	NA	CO_2_-eq emissions
Cecchini, 2010^65^	10% tax on foods high in fat and 10% subsidy on F&V School-based health promotion interventions Worksite health promotion interventions Mass media health promotion campaigns Counseling of individuals at risk in primary care Regulation of food advertising to children Compulsory food labelling	Brazil, China, India, Mexico, Russia, South Africa, England	Healthcare, government	No	No	Obesity, stroke, ischemic heart disease, cancer (lung, colorectal, female breast)	NA
Springmann, 2016^66^	52 USD tax/ton of CO_2_-eq on: All food All food except F&V, legumes, crops Animal-based food Red meat Beef All scenarios above also combined with the revenue used for income compensation All scenarios above also combined with F&V subsidies from 75% of the revenue	150 world regions	Healthcare, environment	Yes	No	Coronary heart disease, stroke, type 2 diabetes, cancer	CO_2_-eq emissions
Revell, 2015^67^	Trend decline in red meat consumption in the developed economy regions 80 USD tax/ton of CO_2_-eq on ruminant meat in developed economies 80 USD tax/ton of CO_2_-eq on ruminant meat Reducing livestock emissions intensities Trend decline in red meat + 80 USD tax/ton of CO_2_-eq on ruminant meat + reducing livestock emissions intensities	World regions: Africa, Asia, Americas and the Caribbean, Europe, Oceania	Environment	No	No	NA	CO_2_-eq emissions
Pearson-Stuttard, 2017^68^	National mass media campaign to increase F&V and reduce SSB consumption 10% tax on SSB 10% subsidy on F&V 30% subsidy on F&V for SNAP participants A combination of these	USA	Healthcare	No	No	Coronary heart disease, stroke, total cardiovascular disease mortality	NA
Blakely, 2020^69^	2 NZD tax on 100 g of saturated fat 0.4 NZD tax on 100 g of sugar 20% subsidy on F&V	New Zealand	Healthcare, consumers	Yes	No	Coronary heart disease, ischemic heart disease, ischemic stroke, hemorrhagic stroke, osteoarthritis, type 2 diabetes, cancers (colorectal, gallbladder, head and neck, kidney, liver, lung, esophagal, pancreatic, stomach, thyroid), female-only cancers: breast, endometrial, ovarian	NA
Veerman, 2013^70^	10% tax on fresh foods, including F&V	Australia	Healthcare	No	No	Ischemic heart disease, ischemic stroke, cancers (lung, esophagus, stomach, colon)	NA
Basu, 2014^71^	Ban on using SNAP dollars for SSBs 30% subsidy on F&V, using SNAP dollars	USA	Healthcare	Yes	No	Obesity, type 2 diabetes	NA
Pitt, 2017^72^	Meat price increase: 5%, 10%, 25%, or 50%	USA	Healthcare	Yes: in sensitivity analysis	No	Obesity	NA
Tiffin, 2011^73^	1% tax on fatty foods for every percent of saturated fat content, maximum 15% tax + subsidy on F&V until revenue neutral	UK	Healthcare	Yes	No	Coronary heart disease, cardiovascular disease, cancers (gastric, lung, overall cancer), ischemic stroke, nontraumatic death	NA
Cash, 2007^74^	1% subsidy on F&V	USA	Healthcare, government	No	No	Ischemic stroke, coronary heart disease	NA
Marshall, 2000^75^	17.5% tax on foods high in saturated fat (whole milk, cheese, butter, pastries, ice cream)	UK	Healthcare	Yes	Yes: calorie stability	Ischemic heart disease	NA
Schroeter, 2008^76^	10% tax on food away from home 10% tax on SSB 10% subsidy on F&V 10% subsidy on diet soft drinks 10% increase in consumer income	USA	Healthcare	Yes	No	Obesity	NA
An, 2015^77^	30% subsidy on F&V (excluding white potatoes, mature legumes, and 100% fruit juice)	USA	Healthcare, government	No	No	All-cause mortality	NA
Dogbe, 2018^78^	Tax on all food: 56 EUR and 200 EUR/ton of CO_2_-eq Tax on meat, fish, dairy, composite dishes: 56 EUR and 200 EUR/ton of CO_2_-eq + subsidy on every other food until revenue neutral	Catalonia (North-East Spain)	Environment, consumers	Yes	No	NA	CO_2_-eq emissions

a The SSCg3d score is a quantitative estimate of how unhealthy a food is.

b Different carbon prices: 42.7, 12.8, or 170.9 GBP/ton CO_2_-eq.

c The WXYfm score is a quantitative estimate of how unhealthy a food is.

Abbreviations: AUD, Australian dollar; COPD, chronic obstructive pulmonary disease; CO_2_-eq, carbon dioxide equivalent; DKK, Danish krone; EBT card, electronic benefits transfer card; EU, European Union; EUR, Euro; F&V, fruits and vegetables; GBP, British pound; GHG, greenhouse gas; GWP100, 100-year global warming potential; N, nitrogen; NA, not applicable; NH_3_, ammonia; NZD, New Zealand dollar; N_2_O, nitrous oxide; P, phosphorus; SNAP, Supplemental Nutrition Assistance Program; SO_2_, sulfur dioxide; SSB, sugar-sweetened beverages; SSCg3d, Scoring system, Group C nutrients, a per 100g base, modification for drinks; UK, United Kingdom; USA, United States of America; USD, United States dollar; VAT, value-added tax; WXYfm, W, X and Y modifications, final model.

### Interventions

The majority of the studies modeled more than 1 intervention (range, 1–32), with high diversity in interventions. In some studies, a combination of interventions was examined; however, fiscal and nonfiscal interventions were seldom looked at together. The most frequently analyzed intervention in our systematic literature review was a subsidy for fruit and vegetables, modeled in 19 studies. The second most common intervention focused on carbon taxes on food, modeled in 17 studies. Carbon taxes on food mean that the tax rate is derived directly from the emissions associated with a given food group. In addition to these 17 studies, 5 others took into consideration, to some extent, the carbon emissions or environmental outcomes of some food groups; however, the tax rate was not derived from carbon emission factors.

Food taxes targeted saturated fat; sugar; salt; full-fat dairy products; processed meat; red meat or meat in general; processed foods; junk food or nonessential, energy-dense food; food consumed away from home; and less healthy foods. Subsidies were targeted to whole grains, legumes, nuts and seeds, fish, seafood, plant oils, meat-substitution products, diet soft drinks, and bottled water. Some studies modeled interventions combining taxes and subsidies on different food groups for the best outcome within the modeling boundaries. Studies have generally assumed that the full tax is passed on to consumers, but 2 studies modeled a pass-through rate of 50% or 60% as scenarios in a sensitivity analysis.^42^^,^^52^

Even though our aim was to study the modeled fiscal interventions on a few predetermined food groups, some studies modeled a range of interventions (fiscal and nonfiscal) besides the ones we were most interested in; for example, a carbon tax on fuels, health promotion, consumer education, food labeling, and regulation of advertising for certain foods. One study, although it modeled interventions, mainly looked at possible future scenarios, such as a declining trend of meat consumption or reduced livestock emission intensity.^67^

The comparator in almost all cases of the studies reviewed was “no new intervention,” meaning that the models compared the new fiscal intervention to current practice as if everything would continue as it is today. In some cases, the authors also modeled some underlying trends as comparators, such as the increase in body mass index (BMI),^72^ cancer incidence,^52^ or red and processed meat consumption.^27^ Also, in 1 case, in a trial-based, cost-effectiveness evaluation, researchers used the collected data after the trial ended as a comparator,^63^ and in another, the “perfect scenario” was the comparator defined as processed meat intake of less than 15 g/day.^45^

### Models

#### General characteristics of the models

The studies were generally country or region specific, with 3 modeling the European Union (EU), 2 modeling a group of countries, and 3 studies modeling almost the entire globe. Most of the models (*n* = 51) used input data from high-income countries; only 4 studies modeled data from middle-income countries.

In line with our exclusion criteria, all studies modeled at least 1 country, but 30 studies did some sort of population stratification and reported outcomes separately for these strata. The usual stratification factors were age, sex, income, socioeconomic status (based on income or education), ethnicity, or, in the case of modeling continents or the world, country-level strata were used.

In 31 studies, researchers modeled health outcomes only; of these, 13 studies used cost-effectiveness analysis, of which 4 were trial-based, cost-effectiveness analyses. Fifteen studies evaluated only the effectiveness, and 2 investigated efficacy and effectiveness as well. In health economics, efficacy means how an intervention works under ideal circumstances, usually in randomized clinical trials, whereas effectiveness means how well the intervention works in everyday practice. Sixteen studies modeled environmental impacts only in the form of an environmental impact analysis. In addition, 8 studies modeled both, of which 7 conducted an environmental impact analysis and an effectiveness evaluation at the same time. Finally, 1 contained a social cost-benefit analysis.

Thirty-five studies used some type of sensitivity analysis. Of these, 11 applied only probabilistic sensitivity analyses, 10 used deterministic sensitivity analysis, and 14 applied both. Only 1 study,^34^ using probabilistic sensitivity analysis, accounted for the covariance between the factors (eg, the correlation between different food intakes).

#### Change in food consumption

In modeling food taxes or subsidies, it is important to capture the change in food consumption conditional on price changes. The central factor in this calculation is called *price elasticity*, which is the percentage change in food consumption in response to a 1% change in food prices. Cross-price elasticities, which are describing food substitutes and complementary foods, were included in 39 models. Price elasticities are of paramount importance in modeling because, in some studies,^26^^,^^54^^,^^71^ estimates were most sensitive to them.

Price elasticity can be calculated from data on food prices and expenditures with different approaches. The source of information on the data varied. Of the 55 articles included, 36 used a nonexperimental econometric method to estimate elasticities based on some applied economic model, 24 used an application of the linear or quadratic Almost Ideal Demand System,^79^ 6 applied approximate Exact Affine Stone Index^80^ Implicit Marshallian Demand system, and 6 used other, less-well-known models such as the Working Preference Independence or Florida model^81^^,^^82^ (all model data can be found in Supporting Information S4). These models are always estimated with an econometric technique under strict assumptions (eg, 3-stage least squares; nonparametric seemingly unrelated regressions; mixed logit model; Heckman correction model). The authors used data from their country or from a different country if data were not available for their own country, preferably adapting from a country with a similar culture.^60^ In 8 instances, researchers used aggregated, country-level or regional data; all other articles reported household or individual-level estimates. Four studies derived price elasticities from experiments, 5 studies from meta-analyses, and 2 studies from both. Additionally, 2 articles borrowed elasticity estimates from a brief review of several articles, 2 used elasticities from a simulation model (International Model for Policy Analysis of Agricultural Commodities and Trade, or IMPACT),^83^ and 4 articles^36^^,^^41^^,^^65^^,^^77^ did not describe in detail how the estimates were obtained.

Six studies^28^^,^^29^^,^^32^^,^^37^^,^^39^^,^^68^ also took into consideration that different socioeconomic groups would have different price sensitivity (usually, those households in which a larger part of the income is spent on food are more responsive to the price change).

The use of inaccurate price elasticities can lead to the under- or overestimation of the health or environmental effect of the fiscal intervention. The accurate measurement of price elasticity is not the only hurdle when modeling changes in consumption. Another concern is the extent to which the amount of food consumed remains constant after a price change; in other words, whether people eat more or less food simply because of the price change. In our review, only 6 studies considered, at least in part, the stability of calorie intake in response to food price changes, and 2 studies considered the weight of the food intake to be constant.^31^^,^^40^

Different methodologies were used for modeling the stability of calorie intake. One model^62^ only allowed liquid calories to change, whereas calories from solid foods remained constant, and 1 study that held the protein intake constant^59^ with the calories.

Studies that have considered calorie stability in the sensitivity analysis^31^^,^^38^ reported that calorie change greatly increases the health effects of a food tax, because a small calorie deficit over time can lead to a significant decrease in obesity rates, and health effects in these models are often linked to obesity. In another study,^57^ which did not impose calorie stability, the authors also expressed doubts about the calorie-change scenarios and believed that the scenario with the highest calorie stability (<1% change) was the most realistic.

Lastly, consumption change and the fiscal interventions inducing this change result not only in a change in consumers’ food expenditure but also in public revenues. In 26 studies, the authors calculated the effect of the tax on the government budget, and in 16 studies, the effect on the consumers and their welfare loss. From the government’s perspective, usually the tax revenue, the subsidy cost, or the implementation cost was modeled; however, in 1 case,^33^ the authors also calculated the revenue change of value-added tax. In 2 cases,^57^^,^^58^ the Gini coefficient was calculated to study the effect of taxation on inequalities.

#### Technical characteristics of the health models

The health models studied in our review can be put into 4 broad categories: state-transition models, comparative risk assessment, attributable risk models, and spreadsheet calculations. There were 19 state-transition models, 10 of these were Markov simulation models, and 9 were Markov cohort models. Ten studies used different types of comparative risk assessment, 4 used an attributable risk model, and in 4 studies, we could only know that it was a spreadsheet calculation because no other specification about the model was mentioned. One study used regression analysis, another performed a utility maximization in a microeconomic framework, and another used back-of-the-envelope calculation.

Nineteen studies did not consider time horizons at all. This means that in their scenarios, the modeled interventions were assumed to have been in place already for a long time, and, therefore, there was no time lag needed for the outcomes to change; the intervention scenario is directly comparable to the no intervention scenario. This was the case with attributable risk models, comparative risk assessments, spreadsheet calculations, and back-of-the-envelope calculations.

Twelve studies used lifetime horizons, and 11 studies had different time horizons. Some studies had several time horizons parallel to 1 another. Of those studies that used a time horizon, nearly all of them used a 3% discount rate; 4 studies did not use a discount rate at all. However, 1 of the latter reported using a 1-year time horizon^61^ only.

Health economic models usually include 1 disease; nevertheless, due to the complex effect of diet on health, many models in this review included more diseases, such as cardiovascular disease in 33 models, obesity in 21 models, type 2 diabetes in 22 models, and cancers in 23 models. Some diseases were rarely included in the models, such as osteoarthritis (*n* = 3 cases), kidney disease (*n* = 2 cases), and underweight (*n* = 2 cases). Finally, chronic obstructive pulmonary disease and liver disease appeared in 1 model only.^62^ One model did not include diseases and just calculated all-cause mortality, and 1 model included nontraumatic death.

#### Technical characteristics of environmental models

The studies usually took the data on the environmental impacts of food from 2 types of sources: 17 studies used existing databases or research articles, and 7 studies computed the environmental impacts specifically for their study.

The geographic coverage of the databases of the environmental impact of food and of the modeling studies themselves usually was the same (in 13 cases). In 8 cases, it was broader than the geographic coverage of the modeling study. Interestingly, 1 had a narrower coverage^54^ (the database covered only some of the EU-15 countries), whereas the study modeled the environmental effect in 27 EU countries. In another study,^58^ the environmental impact data were taken from a different country than the scope of the modeling study. One study did not provide information about the geographic source of the data regarding the environmental impact.^60^

The system boundaries of the environmental impacts are important factors. In other words, to what extent did the studies include the environmental impacts of food production and consumption? These system boundaries or scopes can be classified into the following main groups: (1) farming or primary production, (2) food processing and packaging, (3) storage and distribution, and (4) consumer use, which can be cooking and cooling.^84^

In our review, 3 studies applied a “cradle-to-farmgate” approach, accounting only for the farming or primary production; 3 studies used a “cradle-to-regional distribution center” approach, whereby they also accounted for the food processing, packaging, and transportation to the regional distribution center; 4 studies had a “cradle-to-retail” approach, accounting for the storage and the distribution of the food at the supermarket; 1 study had a “cradle-to-home” approach, accounting for the transport to the place of final consumption; and 7 studies calculated also the cooking of the food, thus taking the “cradle-to-grave” approach.

Three articles reported on studies for which data were obtained from more sources with different system boundaries: cradle-to-farmgate and cradle-to-retail,^27^^,^^66^ or cradle-to-retail and cradle-to-grave. No data were available on the system boundaries in 3 studies^42^^,^^51^^,^^57^; all of them used the same data set computed by a consultancy firm. Some factors were included in the system boundaries of a few studies, including waste management through the whole process, food losses, land-use change related to the primary production, carbon dioxide (CO_2_) release from the soil, change in carbon stocks, transportation of raw materials and products, and consumer travel.

### Outcomes

#### Health outcomes

Of the 55 studies in this review, 39 modeled health outcomes, and 8 of those modeled both health and environmental outcomes. Many different types of health outcome measures were present in the studies, and 1 study regularly used several measures to describe the occurrence of the outcome. BMI, weight change, or change in obesity rates was used 9 times, serum cholesterol was used in 1 study, change in disease risk was used twice, and prevalence or incidence of a disease or of an event was used 10 times. Other types of measures were connected to deaths: life-years saved or lived was used 3 times, the number of deaths or change in mortality rate was used in 8 studies, and measures of deaths prevented or postponed were used in 10. Lastly, in some cases, combined measures were used, which combine disability or quality of life with the life-years: quality-adjusted life years was used 8 times, disability-adjusted life-years was used in 7 studies; and health-adjusted life years was used in 3 studies.

In many models, health outcomes were connected to nutritional outcomes. This was the case in 29 models, whereas in an additional 11 models, only nutritional outcomes were considered without any health outcome. The most usual outcome was energy intake, estimated in 26 studies, whereas 2 studies estimated total dietary weight. Several studies calculated the intake of macronutrients: salt/sodium in 16, sugar in 12, free sugar in 2, carbohydrate in 6, fiber in 11, protein in 9, cholesterol in 10, total fat in 12, saturated fatty acid in 19, monounsaturated fatty acid in 7, and polyunsaturated fatty acid in 8 studies. Interestingly, 2 studies also calculated the amount of plant protein consumed. Micronutrient intakes, like vitamins and minerals, were calculated in 3 studies. Other index-like measures were calculated, such as glycemic load in 1 study, food security index in 1 study, and an index about healthy eating in 4 studies.

In addition to calculating health outcomes, 16 studies also included healthcare costs in their modeling; more specifically, direct healthcare costs were included in all 16 studies and indirect healthcare costs (ie, the cost of treating diseases in the added years of life) were included in 3 studies.^31^^,^^44^^,^^52^ Non-healthcare costs, such as informal care costs and productivity costs, were included in 6 studies.

#### Environmental outcomes

Twenty-four articles modeled environmental outcomes, of which 8 applied both environmental and health outcomes. All 24 studies calculated CO_2_-equivalent emissions, which is a measure of GHG emissions. In addition, several studies estimated other environmental outcomes, such as air acidification measured in sulfur dioxide equivalents (*n* = 4), water eutrophication measured in nitrogen equivalents (*n* = 4), freshwater use (*n* = 2), and land use change (*n* = 2). One study^48^ calculated many other measures. The authors divided the land use change to cropland and pasture use, calculated nitrogen application, phosphorus application, terrestrial extinction rate, nitrogen oxides emissions, ammonia emissions, nitrogen dioxide emissions, and pesticide application.

A few studies (*n* = 4) also estimated the costs associated with future environmental damage and modeled how much the intervention would save in terms of the cost of environmental damage. We could not find any publication in the literature that investigated the impact of the environmental outcomes on health.

## DISCUSSION

There were large differences in the taxation policies and tax rates modeled in the studies included in this review. The models were also very different regarding the outcomes considered and the method of modeling. The differences went far beyond those characteristics we extracted and presented here. We did not find a model that covered all relevant aspects of the issue, because every model misses some part of the full picture. However, reducing the complexity of a problem is an inherent feature of a model, because being overly complex would defeat its original purpose. The model scopes could be visualized as overlapping pieces of a large puzzle. This leads to inherent limitations of each, because they miss important potential effects of the interventions they model. The most comprehensive model incorporated social, environmental, health, and governmental factors and estimated several outcomes using social cost-benefit analysis, but even this model did not use other seemingly important factors like cross-price elasticities or the effect of environmental changes on the health of the population.^33^

### Change in food consumption

The change in food consumption was the central part of all the models. Predicting the population's behavior is seldom easy, especially in terms of food consumption, which is shaped by factors such as personal taste, cultural norms, food commercials, time and skill for preparing the food, and, chiefly, by the price of food.^9^

The reviewed articles presented a wide range of methodologies for obtaining price elasticity estimates. These ranged from very reliable methods that ensure high internal validity, such as regression-adjusted experimental estimates,^30^ to rather arbitrary “expert assumptions.”^75^ Most articles used household or individual data to estimate an applied economic model. These models provide unbiased estimates under some strict assumptions, varying from model to model, such as the (log-)normal distribution or the cross-observational independence of the error terms.

Similar to other health technology assessments, models based on trial data could be the most valid but do not necessarily reflect what could be achieved in real life with the interventions. There were only a few models in which data from a trial were used to calculate the consumption change. In absence of such trials, consumption-change models with a constant price elasticity usually assume that the new dietary pattern will be stable after the price change takes effect. In the absence of longer trials, this assumption was only confirmed by a 1-year study that trialed a 30% subsidy on fruit and vegetables and found a relatively stable consumption pattern.^77^ However, other authors argued that dietary behavior change is hard to maintain; therefore, they calculated, using a 50% decay rate/year, the induced dietary changes.^63^

There are several factors that can influence the accuracy of price elasticity estimation, and these differences are present in the studies included in this review, as well. One factor is the data that serve as input for the price elasticity calculations. Data collected on purchases during a shorter period of time can be unreliable on consumption because households may consume food that was bought earlier or buy food that will be consumed at a later date.^56^ It is also important to know which country's data are used for price elasticity estimation, because consumption patterns and changes depend on culture and standard of living, among other factors.^9^ Also, even when using the modeled country’s data, not every study uses an elasticity estimated for the setting they are modeling. This can also lead to inaccuracies in the modeled consumption, and there may be a need to rescale the price elasticities to the modeled setting.^69^ When price elasticity estimates are not available for a country of interest, the use of data from a country with similar culture might be a solution, with the adjustment of the price elasticities to the current setting in the model.

Price elasticities can differ by socioeconomic groups, according to a meta-analysis.^85^ People in poorer households are more responsive to price changes, and a health-based food tax can lead to greater health effects. Only a few studies modeled this,^28^^,^^29^^,^^32^^,^^37^^,^^39^^,^^68^ but leaving out this aspect can lead to the underestimation of health effects in lower socioeconomic groups.

Furthermore, price elasticity may change over time. Public information campaigns about some foods' health or climate effects can influence price elasticity; thus, the effect of fiscal policies could be augmented.^54^ Only 2 studies modeled an information campaign together with a fiscal policy.^63^^,^^68^ One of these was based on a trial,^63^ and the authors found an additional benefit: the consumers who received the education campaign were more responsive to the price change, namely to the subsidy on fruits and vegetables.^86^ Changing food preferences in a population could be even more important than the fiscal intervention itself, according to some authors.^35^

Another key factor in this context is caloric stability. It is important for modeling, because several models have obesity as an intermediate outcome, and the change in BMI is behind most of their health effects.^44^ Even small changes in consumed calories can lead to body weight differences in the long term, leading to a significant impact on health.^72^ In line with that, other studies also found that the health outcome was very sensitive to the calorie change after the intervention.^38^^,^^69^

There were studies that did not consider the entire calorie intake constant, based on previous evidence. For example, in a systematic review, authors found that increased fruit and vegetable consumption does not result in weight gain; on the contrary, it can cause a small reduction in body weight.^87^ In line with this, Ni Mhurchu et al,^28^ in their modeling study, assumed body weight to remain stable after an increase in fruit and vegetable consumption. Additionally, liquid calories are not satiating,^88^ and 1 of the modeling studies based its calculations on this.^62^

An alternative modeling method to keep the people's weight fairly constant is to assume that the weight of food consumed remains constant after the fiscal intervention^40^ or changes by up to 50 g.^31^ But in these cases, the consumed calories were not stable, so weight changes in the population would still occur in the long term.

Regarding calorie stability or diet weight stability, a meta-analysis showed that people were quite stable on the weight of the diet they ate, and consuming less calorie-dense foods could lead to a weight change.^89^ However, there is evidence suggesting that the picture is probably more complex, with humans also responding to the weight and calorie content of the food they consume.^90^ Until more evidence emerges, the best choice seems to be to use cross-price elasticities and either model a constant weight of consumed food, or be more rigorous and keep the calorie intake constant.

### Health and environment

Besides the food consumption change, the simulated health outcome is heavily influenced by the diseases modeled. In a model that only included obesity as a disease, black men would experience undernutrition and a reduction in life expectancy as a result of a tax on meat.^72^ A similar model, which also modeled tax on meat in the same country, using similar population stratification but taking into consideration other diseases, the outcome was quite different: all population subgroups would experience health gain.^52^

Compared with health effects that will be present in the country of the intervention, the effects on environmental outcomes of food production and consumption may be global.

The studies differed in terms of where the data on the environmental impact of food production were derived from. The advantage of locally calculated data is that they can be differentiated according to the fact that some of the food consumed locally may originate from different locations with different environmental footprints.^91^ Another study’s authors argued that if the tax is introduced in several countries in a region at the same time, it is sufficient to calculate the tax rate based on average emission intensity to reduce administrative costs, because the differences in emissions between the food groups are larger than those between producers.^54^ Even though local data seem to be a better choice, it is not always possible to acquire local data about the emission intensities of foods; in those cases, it is sufficient to use regional data.

It would seem easy to tax the food groups that have the highest emissions and subsidize the foods that have the lowest, but there are studies that show this can have an adverse health effect,^38^^,^^57^ whereas others report only a small health benefit.^62^ This highlights the importance of designing the tax in a way that is beneficial for both health and the environment, because, in some cases, the 2 aims may conflict. or example, there are unhealthy food groups that also have a small carbon footprint, such as sugar.^62^ One approach to address this concern is to consider the protein content of foods and to shift some of the consumption from animal proteins to plant-based proteins,^57^ thus benefiting both population health and the environment.^10^

When designing a climate tax, it is important to take into consideration the already existing taxes and not to duplicate taxation on some of the environmental externalities and fail to tax others, such as land use. One study specifically made an effort to not double tax some environmental externalities, like the CO_2_ emissions connected to fossil fuel use.^47^ This aspect might be usually overlooked, although even the United Nations and the EU stated they wanted to make sure emissions were not double taxed, even though they mostly meant it on the level of international trading.^92^^,^^93^

The system boundaries are a factor to look at when interpreting and comparing the simulated decrease in GHG emissions, because the models are particularly sensitive to them: for example, 1 model concluded that 84%–90% of total emissions reduction was due to land use change, a factor not generally included in the simulation.^62^ With the increasing effects of environmental change already upon us, it seems a better choice to include a wide system boundary and not just to model the usual climate impacts, such as the increase in GHG levels, but also other environmental impacts for our planet has boundaries that we are overstepping.^10^

Interestingly, only 1 study mentioned the health impact of environmental changes,^60^ and neither of the studies in this systematic review estimated it. These health effects can be the consequence of, for example, heatwaves or other extreme weather events. Moreover, when modeling, these effects influence the entire world’s population and not only the modeled region.

### Strengths and limitations

The study has various strengths but also some limitations. A strength of our study is that it focuses on models dealing with both health and environmental models, connecting these 2 topics, which are interconnected in planetary health. The use of rigorous methodology and parallel search and data extraction makes the results more reliable. However, our study has some limitations, as well. The search was limited to 2 databases only; therefore, some studies might have been overlooked, which is an inherent limitation of most systematic reviews, because one needs to find a balance between comprehensiveness and feasibility.

### Similar reviews

In a related review by Emmert-Fees et al,^18^ which aimed to map the simulation studies of population-based dietary policies, the authors reported similar findings. They also found several policies aimed at fruit and vegetables, that the studies rarely evaluated overall diet quality, and the most frequently modeled diseases were similar to those in our review: cardiovascular diseases, diabetes, and cancers. Those authors found that BMI changes are responsible for a significant part of these health models, but they did not discuss the importance of consumption change and calorie stability.^18^ Contrary to our study, Emmert-Fees et al^18^ did not aim to analyze the simulation models regarding environmental effects following dietary change.

## CONCLUSIONS

Although we are already aware of the critical importance of environmental change, and we know roughly what measures should be taken to improve both the environment and people's health, the steps taken so far are not adequate. Well-calibrated, accurate models can help decision-makers and the wider community take actions, because they enable us to predict the consequences of our actions.

We found that none of the reviewed models could fully capture the complexity of the issue at hand. Models need to simplify reality, but there also are disadvantages when they aim to oversimplify our complex world. When important aspects of a phenomenon are missed, the conclusions drawn from the model might be misleading. We still need an appropriate model that covers all relevant elements of the affected areas and their interrelations to estimate the real impact of dietary fiscal policies on societies. From the literature, it is clear that it is preferable to develop a policy package consisting of tax, subsidy, and consumer education working together. The educational and fiscal policies can have multiplicative effects, and the subsidy helps mitigate the regressive effects of the tax.

According to our findings, consumption change, price elasticities, and their accuracy are most important to the validity of the model. Including substitute foods in the model leads to a more accurate picture; failure to take such an aspect into account could lead to the over- or underestimation of the health or environmental effects. Fiscal policies are complex. Sometimes even a seemingly good intervention can have negative effects, and missing factors can make a difference in a model that only partially simulates the intervention.

We still lack a comprehensive model, but in their review, Emmert-Fees et al^18^ designed a logic model of economic evaluations of dietary policies, which can be a starting point to design a model concept of dietary fiscal policies for planetary and human health. It is our firm belief that with more accurate simulation modeling of food taxes and subsidies, the discipline of health economy could contribute to a noticeable positive impact for the people and the planet.

## Supplementary Material

nuae111_Supplementary_Data
