# Supplementary material for: Prospects of Food Taxes for Planetary Health: A Systematic Review of Modeling Studies
Source: Nutr Rev. 2024 Sep 16;83(3):503–24. doi: 10.1093/nutrit/nuae111 (PMC11879128; doi:10.1093/nutrit/nuae111)
Supplement: nuae111_Supplementary_Data [file nuae111_supplementary_data.docx]

# Search details

### The search string for Pubmed was the following:

("food pric*"[Title/Abstract] OR "tax"[Title/Abstract] OR "subsid*"[Title/Abstract] OR "fiscal*"[Title/Abstract] OR "taxation"[Title/Abstract] OR "taxes"[Title/Abstract] OR "taxing"[Title/Abstract] OR "price discount*"[Title/Abstract])

AND

("vegetable*"[Title/Abstract] OR "fruit*"[Title/Abstract] OR "meat*"[Title/Abstract] OR "dair*"[Title/Abstract] OR "animal product*"[Title/Abstract] OR "food*"[Title/Abstract] OR "Dairy Products"[MeSH Terms] OR "whole grain*"[Title/Abstract] OR "fish*"[Title/Abstract] OR "egg"[Title/Abstract] OR "eggs"[Title/Abstract] OR "nut"[Title/Abstract] OR "nuts"[Title/Abstract] OR "legume*"[Title/Abstract])

AND

(("cost benefit analysis"[MeSH Terms] OR "technology assessment, biomedical"[MeSH Terms] OR "model*"[Title/Abstract] OR "environmental impact assessment"[Title/Abstract] OR "environmental assessment"[Title/Abstract] OR "life cycle analysis"[Title/Abstract] OR "cost-benefit"[Title/Abstract] OR "cost effectiv*"[Title/Abstract] OR "health technology assessment"[Title/Abstract] OR "cost-utility"[Title/Abstract] OR "scenario*"[Title/Abstract]) OR "multistate life-table"[Title/Abstract] OR "comparative risk assessment"[Title/Abstract])

AND

("Health Care Costs"[MeSH Terms:noexp] OR "Cost of Illness"[MeSH Terms] OR "Global Burden of Disease"[MeSH Terms] OR "Quality-Adjusted Life Years"[MeSH Terms] OR "Carbon Footprint"[MeSH Terms] OR "Greenhouse Gases"[MeSH Terms] OR "qaly*"[Title/Abstract] OR "quality adjusted life year*"[Title/Abstract] OR "health adjusted life year*"[Title/Abstract] OR "disability adjusted life year*"[Title/Abstract] OR "daly*"[Title/Abstract] OR "haly*"[Title/Abstract] OR "DPP"[Title/Abstract] OR "mortalit*"[Title/Abstract] OR "health gain*"[Title/Abstract] OR "cost saving*"[Title/Abstract] OR "health cost*"[Title/Abstract] OR "healthcare cost*"[Title/Abstract] OR "health care cost*"[Title/Abstract] OR "disease burden*"[Title/Abstract] OR "greenhouse gas*"[Title/Abstract] OR "GHG"[Title/Abstract] OR "CO2"[Title/Abstract] OR "environmental impact*"[Title/Abstract] OR "footprint*"[Title/Abstract] OR "carbon dioxide"[Title/Abstract] OR "emission*"[Title/Abstract] OR "climate friendl*"[Title/Abstract])

### The search string for Scopus was the following:

TITLE-ABS-KEY ( "tax*" OR "fiscal*" OR "subsid*" OR "food pric*" OR "price discount*" )

AND

TITLE-ABS-KEY ( "vegetable*" OR "fruit*" OR "meat*" OR "dair*" OR "animal product*" OR "food*" OR "whole grain*" OR "fish*" OR "egg*" OR "nut*" OR "legume*" )

AND

TITLE-ABS-KEY ( "model*" OR "environmental impact assessment" OR "environmental assessment" OR "life cycle analysis" OR "cost-benefit" OR "cost effectiv*" OR "health technology assessment" OR "cost-utility" OR "scenario*" OR "multistate life-table" OR "comparative risk assessment" )

AND

TITLE-ABS-KEY ( "qaly*" OR "quality adjusted life year*" OR "health adjusted life year*" OR "disability adjusted life year*" OR "daly*" OR "haly*" OR "DPP" OR "mortalit*" OR "health gain*" OR "cost saving*" OR "health cost*" OR "healthcare cost*" OR "health care cost*" OR "disease burden*" OR "greenhouse gas*" OR "GHG" OR "CO2" OR "environmental impact*" OR "footprint*" OR "emission*" OR "climate-friendl*" OR "carbon dioxide" )

### Search details for gray literature searches

OpenDissertations

- search query: food AND tax AND subsidy AND price

Dart-Europe:

- search query: food AND tax
- language: english, hungarian
